# Chinese Medicine Enhancing Response Rates to Immunosuppressant PD-L1 Inhibitor and Improving the Quality of Life of Hepatocellular Carcinoma-Bearing Mice

**DOI:** 10.5812/ijpr-134216

**Published:** 2023-04-07

**Authors:** Lixing Liu, Hao Li, Peijin Li, Rui Zhou, Qinglin Zhang, Tingting Liu, Li Feng

**Affiliations:** 1 Department of Chinese Medicine, National Cancer Center, National Clinical Research Center for Cancer, Cancer Hospital, Chinese Academy of Medical Sciences and Peking Union Medical College, Beijing, China; 2 National Cancer Center, National Clinical Research Center for Cancer, Hebei Cancer Hospital, Chinese Academy of Medical Sciences, Langfang, China; 3 Dongzhimen Hospital, Beijing University of Chinese Medicine, Beijing, China

**Keywords:** Hepatocellular Carcinoma, Immune Checkpoint Inhibitors, Chinese Medicine, Tumor Micro-environment

## Abstract

**Background:**

Malignant tumors are a significant disease endangering human health. Chinese Medicine (CM) plays an important role in comprehensive and holistic tumor treatment.

**Objectives:**

We aimed to investigate whether CM combined with the immunosuppressant PD-1/PD-L1 inhibitor has a good synergistic effect and can significantly improve response rates for the immunosuppressant.

**Methods:**

We combined CM with immunosuppressant in treating six-week-old hepatocellular carcinoma-bearing mice and compared the outcomes of groups undergoing different interventions: blank group, control group, CM group, PD-L1 inhibitor group, and CM + PD-L1 inhibitor group, with ten mice in each group. The quality of life was evaluated along with the tumor inhibition effects and growth rates.

**Results:**

CM significantly reduced tumor load and improved the quality of life of cancer-bearing mice. The survival rate was 81.8% in the control group, 100% in the CM group, 90.9% in the PD-L1 inhibitor group, and 100% in the combined group in the first week. The survival rate was 45.5% in the control group, 54.5% in the CM group, 81.8% in the PD-L1 inhibitor group, and 81.8% in the combined group in the second week. 38% mice in the CM+PD-L1 inhibitor group with smaller tumor size than the average of the control group, which was much higher than other treatment groups. CM also reduced the expression of JAK2 mRNA and STAT3 mRNA, although not significantly (P > 0.05), and reduced PD-L1 mRNA in tumor tissue compared to the control group (P < 0.05).

**Conclusions:**

CM had a synergistic effect on PD-L1 inhibitors and increased response rates to PD-L1 inhibitor treatment.

## 1. Background

Malignant tumors are a significant disease endangering human health. In recent years, the incidence rate of malignant tumors has shown an upward trend in China. The global 5-year survival rate of liver cancer is 5 - 30% (1), and in China, it is one of the tumors with the lowest 5-year survival rate. According to the National Cancer Center, the incidence of liver cancer in China is 26.92/100000, ranking fourth among the incidence rates of all cancers, and mortality due to liver cancer is 23.72/100000, ranking second among the causes of cancer death (2). Moreover, the incidence and deaths due to liver cancer in China account for approximately half of the worldwide cases (3), and there is an urgent need for effective treatment schemes and drugs. Immunosuppressant PD1/PD-L1 inhibitors bring new hope to tumor patients. Still, the proportion of responders to these treatments is lower among patients with liver cancer than among those with other tumors, with more than 70% of liver cancer patients not benefitting from the treatment.

Chinese Medicine (CM) plays an important role in comprehensive and holistic tumor treatment (4, 5). According to preliminary clinical statistics, about 70% of tumor patients receive different treatments that fall under CM in China (6). We propose taking full advantage of CM in cancer prevention, treatment, symptom relief, and rehabilitation to prolong overall survival and improve quality of life. CM has the advantage of regulating the patient's "internal environment" and enhancing immunity (7, 8). A meta-analysis shows that CM combined with radiotherapy and chemotherapy can significantly prolong survival times, improve quality of life, and reduce the toxic and side effects of radiotherapy and chemotherapy (9).

Immunosuppressants have become increasingly important in treating hepatocellular carcinoma, and several clinical trials have been conducted, including nivolumab and pembrolizumab (10-12). In a randomized, double-blind trial comparing pembrolizumab with a placebo in hepatocellular carcinoma, OS and PFS did not meet the preset plan (13). Meanwhile, immune-related adverse events (IRAE) gradually increase after anti-CTLA-4 and anti-PD-1 therapy. Studies (14-18) showed a 59% incidence of Grade 3 or 4 IRAE in the combination of nivolumab and ipilimumab group, compared with 28% in ipilimumab and 21% in nivolumab. The most common grade 3 or 4 IRAE are gastrointestinal reactions, fatigue, and itching. Studies have found that CM combined with immunosuppressants can play a better role in anti-tumor (19). Zhang et al. found that CFF-1 could not only inhibit PD-1/PD-L1 but also prolong the survival time of the mouse model of metastatic prostate cancer and block lung metastasis (20). However, no studies have been conducted on treating hepatocellular carcinoma by CM combined with immunosuppressive.

## 2. Objectives

Here, we aimed to investigate whether CM combined with the immunosuppressant PD1/PD-L1 inhibitor has a good synergistic effect and whether it can significantly improve response rates to the immunosuppressants. To that end, we conducted an exploratory animal study on the efficacy and mechanism of CM combined with immunosuppression in treating Hepatocellular carcinoma.

## 3. Methods

### 3.1. Animal

Six-week-old male ICR mice were purchased from Beijing Huafukang Biotechnology Co., Ltd, Beijing, China. The mean animal weight was 22 ± 2 g. The animals were raised in a specific pathogen-free environment at room temperature (22°C) and relative humidity of 50%, with lights on between 06:00 to 18:00 hours. The mice were provided food and water ad libitum; each cage (25 × 15 × 18 cm^3^) housed five mice. The study received ethical approval from the experimental animal center of the Cancer Hospital of the Chinese Academy of Medical Sciences (the ethical approval code: XYXKJing2019-0019).

### 3.2. Hepatocellular Carcinoma Mice Model

The H22 mouse hepatoma cells (purchased from Cobioer Biosciences, Co., Ltd, Nanjing, China) were inoculated into the abdominal cavity of ICR mice under sterile conditions to form ascites tumors to harvest more H22 mouse hepatoma cells. After two to three weeks of growth, mice ascites tumor cells were obtained under sterile conditions. And then, they were diluted with normal saline and counted to make a tumor cell suspension with a concentration of 1×10^8^ cells/mL. The subcutaneous inoculation amount was 0.2 mL/mouse on the back of the right forelimb. According to the tumor size evaluation formula: long × Width 2 × π/6, the tumor size was measured with a vernier caliper and recorded daily. Fifty mice with tumor tissue of 150 - 300 mm^3^ were selected and transferred to experiments (Table 1).

**Table 1.** Grouping and Intervention

Group	HCC Model	Intervention	Administration Way	Dosage	Frequency
**Blank group**	No	Water	i.g.	0.1 mL	Four days a week
**Control group**	Yes	Water	i.g.	0.1 mL	Four days a week
**CM group**	Yes	Chinese medicine decoction	i.g.	1.65 g/kg	Four days a week
**PD-L1 inhibitor group**	Yes	PD-L1 inhibitor	i.v.	12.5 mg/kg	Once a week
**CM+PD-L1 inhibitor group**	Yes	Chinese medicine decoction & PD-L1 inhibitor	i.g.+i.v.	1.65 g/kg + 12.5 mg/kg	CM: Four days a week; PD-L1 inhibitor: Once a week

### 3.3. Grouping

Fifty mice were randomly divided into five groups with ten mice in each: blank group, control group, CM group, PD-L1 inhibitor group, and CM + PD-L1 inhibitor group. The CM intervention group was administered CM (Chinese medicine prescription containing Baishao, Shanyao, etc.) daily at a dose of 1.65 g/kg four days a week (an oral drug for four days, rest for three days) and with a total of two weeks of treatment. PD-L1 inhibitor (purchased from Bio X Cell, Lebanon, NH, USA, BE0101) was administered via tail vein intravenous injection once weekly at 12.5 mg/kg for 14 days. The control group was orally administered water (Table 1).

### 3.4. Observation Indices

The mice were sacrificed after two weeks of CM. Half of the tumor and spleen samples were soaked in 4% paraformaldehyde phosphate buffer solution, and fixed, while the remaining tissue of the tumor and spleen were frozen in liquid nitrogen. Quality of life (21): The weight, posture, mobility, eating behavior, and hair of tumor-bearing mice were evaluated every day to assess their quality of life. Tumor inhibition effects were assessed every day by regularly evaluating changes in the tumor volume in each group with a vernier caliper, using the following formula: length × width 2 × π/6. The tumor growth rate was calculated every day by comparing the tumor volume with that at the baseline. Survival observation (22): After the interventions in each group, survival rates within 14 days of the four tumor-bearing groups of mice were observed, the time of death (in days) was recorded, and a survival curve for the tumor-bearing mice was generated.

### 3.5. Real-time Quantitative Polymerase Chain Reaction (RT-qPCR)

Real-time PCR was performed using PowerSYBR Green PCR Master Mix (Applied Biosystems, CAS No: 19911596) following the manufacturer’s instructions. The primer sequences were designed on ncbi-primer (Table 2). Β-actin was used as an internal control. Relative quantitative analysis was conducted by 2^-ΔΔct^ methods.

**Table 2.** Primers

Primer	Sequence
**β-actin-F**	CTTCCAGCCATCCTTCTTG
**β-actin-R**	CGGTGATTTCCTTCTGCATT
**JAK2-F**	TCTGTGGGAGATCTGCAGTG
**JAK2-R**	CACGGATGACAGCTCTGAAA
**STAT3-F**	GACCCGCCAACAAATTAAGA
**STAT3-R**	TCGTGGTAAACTGGACACCA
**PD-L1-F**	CGCCTCACTTGCTCATTACA
**PD-L1-R**	CTGTCAAGGGCTCACACAGA

### 3.6. Statistical Analysis

The SPSS 11.5 statistical analysis software (IBM, Armonk, NY) was used for data analyses. The data are expressed as mean ± standard deviation. Differences were considered statistically significant at P < 0.05. One-way ANOVA was used for comparisons between multiple groups, and *t*-tests were used for comparisons between two groups.

## 4. Results

### 4.1. CM Improving the Quality of Life of Tumor-bearing Mice

**Figure 1.**
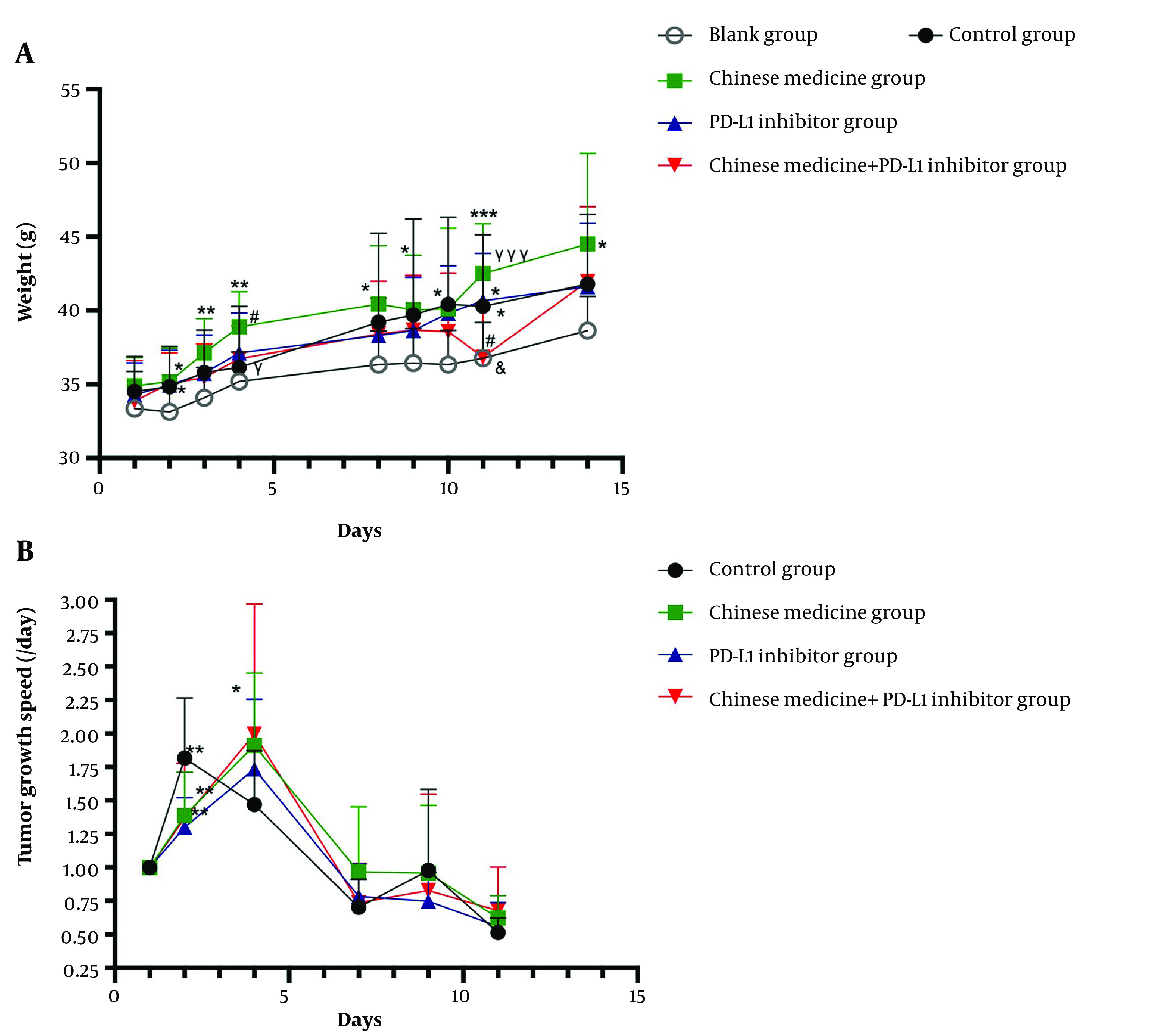
A, Weight changes in all groups. B, Tumor growth rates in each tumor-bearing mice group. * Compared with the Blank group; # compared with the Control group; γ compared with the Chinese medicine group; & compared with the PD-L1 inhibitor group. *P ≤ 0.5, **P ≤ 0.01, ***P ≤ 0.001 (similarly hereinafter).

With the growth of tumor tissue, the weight gain of each tumor-bearing mice group was significantly higher than that of the non-tumor-bearing mice group. The average daily weight gain of the control group was 0.77 g, while the average daily weight gain of the blank group was 0.38 g. The tumor growth was accompanied by reduced activity and food intake and dry, withered, towering, and sparse hair. The CM group exhibited strong vitality, more food consumption, soft and glossy hair, and higher weight than the control and PD-L1 inhibitor groups, with the average daily weight gaining 0.98 g in the CM group and 0.80 g in the PD-L1 inhibitor group. This difference became more evident as the disease progressed. The weight changes of the mice in each group are shown in Figure 1A. The mice in the CM group gained significantly more weight than the control group mice (P < 0.05 or P < 0.01). There was also a statistically significant weight difference between the CM and CM + PD-L1 inhibitor groups on the 4th and the 11th day, respectively (P < 0.05 and P < 0.001). However, there was no significant difference between the CM and PD-L1 inhibitor groups or between the CM + PD-L1 inhibitor and control groups, indicating that CM and immunosuppressant treatment could improve the quality of life of mice and enhance the anti-tumor growth effects of immunosuppressant (Figure 1A).

### 4.2. CM Reducing Tumor Growth Rates in Tumor-bearing Mice in the Early Tumor Stage

During the observation period, changes in the tumor volume were monitored. We took the tumor volume measured on the first day of the observation period as the benchmark and then measured the tumor volume ratio to the first day divided by the growth days to get the tumor growth rate. Our observations showed that tumor growth could be inhibited during the first week and that improvements in the quality of life of tumor-bearing mice were mainly visible during the second week. There was a significant statistical difference in tumor growth rate between the CM and control groups (P < 0.01) in the first week (Figure 1B), and there were no significant statistical differences between the treatment groups (P > 0.05) (Figure 1B). CM combined with PD-L1 inhibitor significantly reduced the tumor volume of most mice, suggesting an increase in the response rate to the PD-L1 inhibitor.

### 4.3. CM Combined with PD-L1 Inhibitor Significantly Prolonged Survival Times and Survival Rates

**Figure 2.**
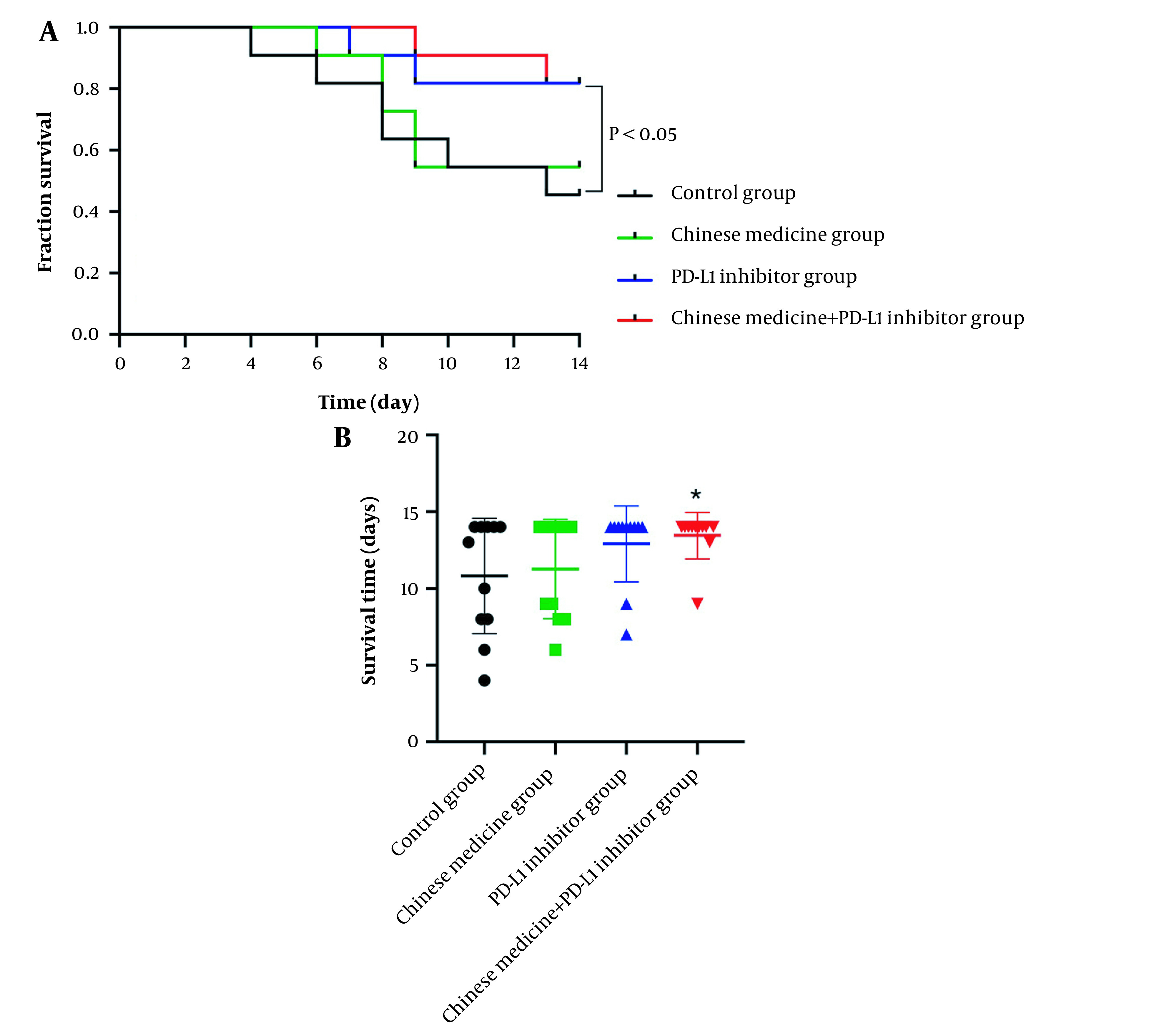
Survival times and survival rates in each tumor-bearing mice group.

The survival times of the tumor-bearing mice in each group were observed. The survival curve showed that the number of deaths in the control group gradually increased (Figure 2A). The survival rate was 81.8% in the control group, 100% in the CM group, 90.9% in the PD-L1 inhibitor group, and 100% in the combined group at the first week. The survival rate was 45.5% in the control group, 54.5% in the CM group, 81.8% in the PD-L1 inhibitor group, and 81.8% in the combined group in the second week. The median survival time of the control group was 13 days. Still, at the end of the observation period, no median survival time could be observed in the PD-L1 inhibitor and the combined CM group (Figure 2A). This showed that CM combined with immune agents could significantly prolong survival times and the difference was significant between CM combined with PD-L1 group and the control group (P < 0.05, Figure 2B).

### 4.4. Synergistic Effects of CM on PD-L1 Inhibitors

**Figure 3.**
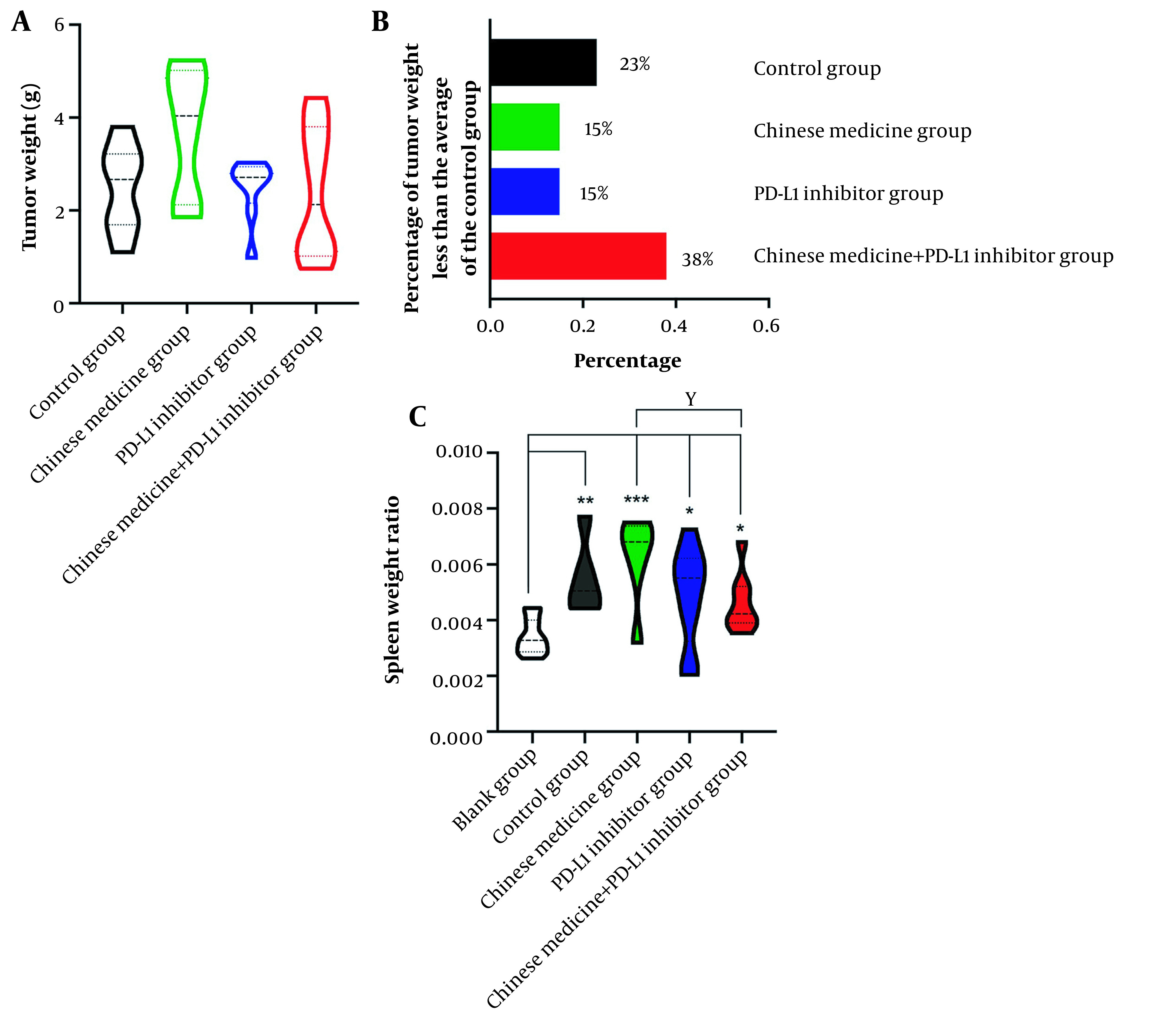
A, Tumor weight in each tumor-bearing mice group. B, The percentage of tumor weight in the treatment groups that is less than the control group's average. C, The spleen organ ratio of each tumor-bearing mice group.

After 14 days of treatment with CM and PD-L1 inhibitors, the proportion of mice with reduced tumor weight in the CM + PD-L1 inhibitor group was significantly higher than that in the single PD-L1 inhibitor group. At the same time, the mice with a large tumor load survived well. The average tumor weight in the control group was 2.49 g. The percentage of tumors with a weight below the average was 23% in the control group, 15% in the CM group, and 15% in the PD-L1 inhibitor group. CM combined with PD-L1 inhibitor could significantly improve the anti-tumor effects of the treatment, with the percentage of tumors below the average weight in the CM + PD-L1 inhibitor group way above that in all other groups, at 38% (Figure 3A-B).

### 4.5. CM Reducing the Spleen Organ Ratio of Tumor-bearing Mice Close to that of Normal Mice

The spleen is the largest and most efficient immune organ in the body. It is the center of cellular and humoral immunity and plays a role in immune monitoring, regulation, and filtration. The main cell components in the spleen are B-lymphocytes, T-lymphocytes, and macrophages, among others. The spleen organ ratio in our mice increased significantly in all intervention groups compared with the control group (P < 0.05, P < 0.01, and P < 0.001), while there was no significant difference between the CM group and PD-L1 inhibitor group (P > 0.05) or between the PD-L1 inhibitor group and combined drug group (P > 0.05). The spleen organ ratio in the combined drug group decreased compared with that in the CM group (Figure 3C).

### 4.6. CM and PD-L1 Inhibitors Reducing the mRNA Expressions of the Key Molecules of the JAK2/STAT3 Pathway

**Figure 4.**
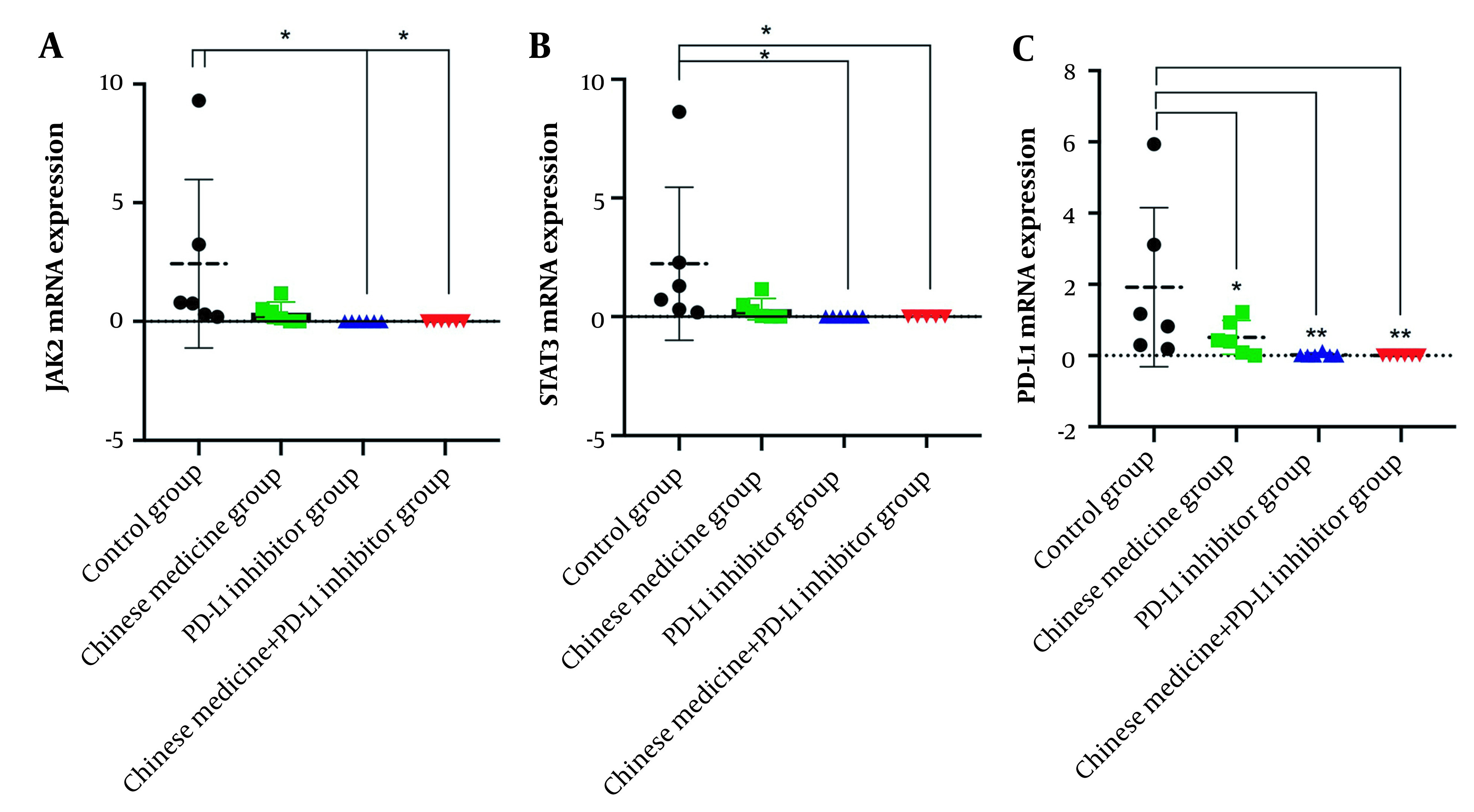
A-C, The mRNA expressions of key molecules of the JAK2/STAT3 pathway in each tumor-bearing mice group.

The expression of JAK2 mRNA in tumor tissue in the control group was significantly increased. The CM group showed reduced expression levels of JAK2 mRNA (although the difference was not statistically significant; P > 0.05). The expression of JAK2 mRNA in the PD-L1 inhibitor group and the CM + PD-L1 inhibitor group was also significantly reduced. The difference with the control group was statistically significant (P < 0.05) (Figure 4A). The expression of STAT3 mRNA in tumor tissue in the control group was significantly increased, while it was reduced in the CM group (although the difference was not statistically significant; P > 0.05) and significantly reduced in the PD-L1 inhibitor and CM + PD-L1 inhibitor groups (compared to the control group; P < 0.05) (Figure 4B). The expression of PD-L1 mRNA in tumor tissue in the control group was significantly increased and, compared to the control group, lower in the CM group (P < 0.05), PD-L1 inhibitor group (P < 0.01), and CM + PD-L1 inhibitor group (P < 0.01) (Figure 4C).

## 5. Discussion

Studies reported that Chinese medicine played a unique role in improving symptoms and quality of life (23-25). Significantly as the process of tumor development, when the tumor load gradually increases, the consumption of the host also gradually increases, and the quality of life in all aspects, such as emaciation, loss of appetite, fatigue, poor nutritional status, poor spirit, physical strength, and decreased activity tolerance is decreased. Chinese medicine significantly improves this status, which is consistent with the performance of tumor-bearing mice observed in our experiment. In the present study, the tumor-bearing mice showed a significant decrease in activity, decreased food intake, and dry hair in the second week. In contrast, after the intervention of Chinese medicine, they showed a significant increase in appetite, improved hair and nutritional status, increased activity, and physical strength.

In the theory of Chinese medicine, prevention is more important than treatment. Some studies (26-28) have found that the intervention of Chinese medicine in the early stage to adjust the body constitution can help improve the symptoms of some chronic diseases and the possibility of disease progression. This study showed that Chinese medicine could inhibit tumor growth rate at the early stage, which was worth considering. The design of the primary study was to start the intervention of Chinese medicine after the tumor cells grew to 150-300m3 after the tumor cells were planted. However, whether the intervention in advance, such as the intervention of Chinese medicine while the tumor cells are inoculated, whether a better performance of Chinese medicine in inhibiting tumor growth could be developed.

PD-L1 inhibitor treatment mainly improves T cell-mediated immune responses in the tumor microenvironment to recognize and kill tumor cells. However, anti-PD-1/PD-L1 immunotherapy is unsatisfactory, with response rates between 20% and 30% (29). The CheckMate 040 phase I/II clinical trial of PD-1 inhibitor in the treatment of primary hepatocellular carcinoma reported response rates between 15% and 20% (10, 30). The main reasons for these low response rates of immunosuppressants are mutations affecting the immunogenicity of the tumor itself, variability in immune checkpoint ligand expression, and the reduction of T cell infiltration, resulting in PD-1 inhibitor resistance. Improving the efficacy of PD-1/PD-L1 blockade is thus the main challenge. In this study, the combination of Chinese medicine and PD-L1 inhibitor could enhance the inhibitory effect of PD-L1 on tumor growth. Chen et al. found that combining Pien Tze Huang (PZH) and PD-1/PD-L1 antibody could slow down tumor growth and improve the infiltration and function of CD8+ T cells compared with mono-therapy, which showed that PZH had a synergistic enhancement effect on immunotherapy (31). These findings were consistent with our studies. The present study showed that the Chinese medicine and PD-L1 inhibitor group prolonged survival times and increased survival rates to nearly twice those observed in the control group. The tumor reduction ratio increased to 250% compared with that of PD-L1 inhibitor alone, significantly improving the response rate of PD-L1 inhibitor. At present, the mechanism of this synergy is still unclear. However, in previous studies, it has been reported that Chinese medicine could regulate immune function (32-34). In the primary research, we found that the ratio of the spleen to organ increased after the intervention of Chinese medicine, while since the spleen contains many immune cells, the intervention of Chinese medicine may regulate the immune function. However, it is regrettable that the spleen immune cell classification test has not been carried out in this study, which will be further improved in future experiments.

Hepatocellular carcinoma patients have higher immune tolerance when treated with PD-L1, and PD-L1 blockade does not significantly prolong the survival of hepatocellular carcinoma patients compared with other tumor entities, suggesting that blocking PD-L1 / PD-1 axis alone may not be sufficient to initiate an adequate level of anticancer immunity in hepatocellular carcinoma (35, 36). Interestingly, STAT3 may directly bind to the PD-1 promoter and activate PD-1 protein expression in T cells (37). Recent studies have shown that inhibition of STAT3 can reduce the expression of PD-L1, thereby inhibiting tumor inflammatory response and improving the immune response to tumor cells (38, 39). Therefore, inhibition of STAT3 signal transduction alone or combined immunotherapy may improve patient prognosis (40). The primary study showed that Chinese medicine significantly reduced the expression of JAK2, STAT3, and PD-L1 mRNA, through which Chinese medicine may function synergetically with PD-L1 inhibitors.

In the present study, CM could significantly reduce the tumor growth rate in the early tumor stage, enhance the response to immunosuppressant PD-L1 inhibitor, exert a synergistic effect, and enhance the effect of the PD-L1 inhibitor attacking the tumor. It also improved the diet and activity levels of mice with a large tumor load to improve their quality of life. CM could significantly increase the total reserve of spleen cells and reduce the expression of JAK2 and STAT3. However, because the regulation of spleen cells is a complex process, it is necessary to clarify further the pathway and mechanism of action of CM. The following potential issues need to be considered when interpreting the results of this study. CM and immunosuppressant treatments were applied for 14 days after tumor inoculation, and the CM group showed obvious inhibition of tumor growth in the early stage. Hence, whether the earlier application of CM treatment on the day of model establishment, for example, CM, may show a better performance of enhancing the anti-tumor effect remains to be researched. The regulatory effect of CM on inflammatory factors and immune cells in the hepatocellular carcinoma microenvironment is also worthy to further study.

### 5.1. Conclusions

The current study demonstrates two functions of CM: firstly, it enhanced the response rate to immunosuppressant PD-L1 inhibitor, which led to a synergistic effect and enhanced the effect of PD-L1 inhibitor on the tumor; secondly, CM improved the diet and activity of mice with a large tumor load, which further improved their quality of life.
